# Soil Microbial Communities Altered by Titanium Ions in Different Agroecosystems of Pitaya and Grape

**DOI:** 10.1128/spectrum.00907-21

**Published:** 2022-02-02

**Authors:** Yuan He, Xin-Yi Hou, Cai-Xia Li, Yan Wang, Xin-Rong Ma

**Affiliations:** a Chengdu Institute of Biology, Chinese Academy of Sciences, Chengdu, China; b University of Chinese Academy of Sciences, Beijing, China; USDA - San Joaquin Valley Agricultural Sciences Center

**Keywords:** soil microbial communities, titanium ions, pitaya, grape, Panxi areas, Panzhihua and Xichang areas

## Abstract

Titanium (Ti) is an element beneficial to plant growth. Application of titanium to roots or leaves at low concentrations can improve crop yield and performance. However, the effect of titanium ions on the bulk soil microbial community of planted crops remains unclear. This study aimed to explore the effects of titanium on soil bacterial and fungal communities. Field surveys were conducted to determine the effect of titanium ions on bulk soil microbial communities in pitaya and grape plantations of Panzhihua and Xichang areas, respectively. Full-length 16S rRNA and internal transcribed spacer (*ITS*) amplicon sequencing were performed using PacBio Sequel to further explore the composition and structure of soil microbiota. The application of titanium ions significantly altered the composition and structure of soil microbiota. Root irrigation with titanium ions in pitaya gardens reduced the diversity of soil fungi and bacteria. However, the decline in bacterial diversity was not statistically significant. Meanwhile, foliar spray of titanium ions on grapes greatly reduced the soil microbial diversity. The bulk soil microbiota had a core of conserved taxa, and titanium ions significantly altered their relative abundances. Furthermore, the application of titanium increased the interaction network of soil fungi and bacteria compared with the control group. Thus, titanium ions potentially improve the stability of the soil microbial community.

**IMPORTANCE** Pitaya and grape are important cash crops in the Panzhihua and Xichang areas, respectively, where they are well adapted. Titanium is a plant growth-promoting element, but the interaction between titanium and soil microorganisms is poorly understood. Titanium ions are still not widely used for growing pitaya and grape in the two regions. Thus, we investigated the effects of titanium ions on soil microbial communities of the two fruit crops in these two regions. Microbial diversity decreased, and the community structure changed; however, the addition of titanium ions enhanced cooccurrence relationships and improved the stability of the community. This study provides a basis for the importance of titanium ion application in crop cultivation.

## INTRODUCTION

The key to meeting the growing global population demand is to improve the efficiency of food production while reducing the negative impact on the environment (1, 2). This can only be achieved by adopting sustainable and eco-friendly agricultural methods, such as applying new growth regulators, maintaining soil microecological balance, and incorporating probiotics (3–5).

Microbes are closely related to plants, play key roles in plant growth and development, and could establish close associations with plants (6). They boost soil nutrient status by decomposing organic matter and fixing nitrogen. Additionally, microbes promote nutrient absorption from the soil (such as nitrogen, phosphorus, and potassium), secrete plant hormones to promote plant growth, and produce metabolites to help plants resist biotic and abiotic stresses (7–9). Despite their importance to plants, our knowledge of microbial interaction with plant growth and development is still minimal (10, 11). To harness agricultural microbes for food security, it is necessary to comprehensively study how agricultural systems (crops and tillage practices) interact to influence microbial communities (9, 12, 13).

Titanium (Ti) is a transition metal that occurs mainly as ilmenite and rutile. It is widely distributed in the crust and lithosphere, typically existing in the tetravalent form. Titanium has two valence states of Ti^4+^ and Ti^3+^, which convert light into chemical energy and trigger redox reactions (14). Although Ti is not an essential element for plant nutrition, its growth-promoting effect on plants has been recognized (15). Studies have shown that titanium ions can promote crop growth and development by stimulating specific enzyme activities and increasing chlorophyll content and photosynthesis (16). A commercial fertilizer containing titanium, Tytanit, can improve crop yield and quality (17). Additionally, titanium can degrade organic pesticides and herbicides in soil by redox reactions and photocatalysis (18); it can also inhibit bacterial growth and extend the vase life of cut flowers (19). However, the effects of titanium ions on the soil microbial community remain unclear.

Panzhihua and Xichang areas are collectively referred to as the Panxi region in Sichuan Province, China. The Panxi area has vast mountains and abundant light and heat resources in the river valley, which are suitable conditions for agriculture. The area mainly has red soil, which is slightly acidic, with low soil-base saturation. However, the organic matter content of the soil is rich in nitrogen and potassium but lacks phosphorus (20).

Pitaya (Hylocereus polyrhizus sp.) is a novelty fruit with high nutrient content and drought tolerance. It is cultivated in tropical or subtropical areas (21, 22). Pitaya is one of the most economically important crops in Panzhihua city. The area offers a suitable climate for pitaya cultivation with fewer diseases, freezing, and typhoons than other Pitaya-producing areas in China.

Crimson seedless grape (Vitis vinifera L.) is popular with consumers worldwide because of its unique crisp and sweet taste (23, 24). It is an economically important crop in the Xichang area, with a climate suitable for its growth. The grape variety can be planted at a high planting density in the Xichang area and is associated with high yield and good economic benefits. These two important fruits were first used in a pilot study, in which the application of titanium ions was tested in two orchards.

This study aimed to elucidate the role of titanium ions in promoting plant growth in relation to soil microorganisms. Field surveys were conducted to examine whether the application of titanium ions to crops would affect soil microbial composition and diversity. PacBio Sequel full-length amplicon sequencing analysis was performed to explore soil microbial composition and diversity. The findings of this study will provide a basis for the application of titanium ions in crop cultivation.

## RESULT

### Overview of the of bacterial and fungal diversity in soil samples.

A total of 170,115 and 192,199 circular consensus sequences (CCSs) were obtained from the 16S rRNA and internal transcribed spacer (*ITS*) gene sequencing of the four groups of samples using the PacBio Sequel. Of these, 143,646 and 183,025 effective CCSs were obtained after identifying the PacBio barcode sequences, quality filtering, and removing chimeras. Each bacterial sample had sequences ranging from 3,790 to 7,058, with a median of 6,684 and an average read length of 1,457 bp (median of 1,457 bp). The sequences from each fungal sample ranged from 7,057 to 7,831, with a median of 7,669 and an average read length of 631 bp (median 620 bp).

**FIG 1:**
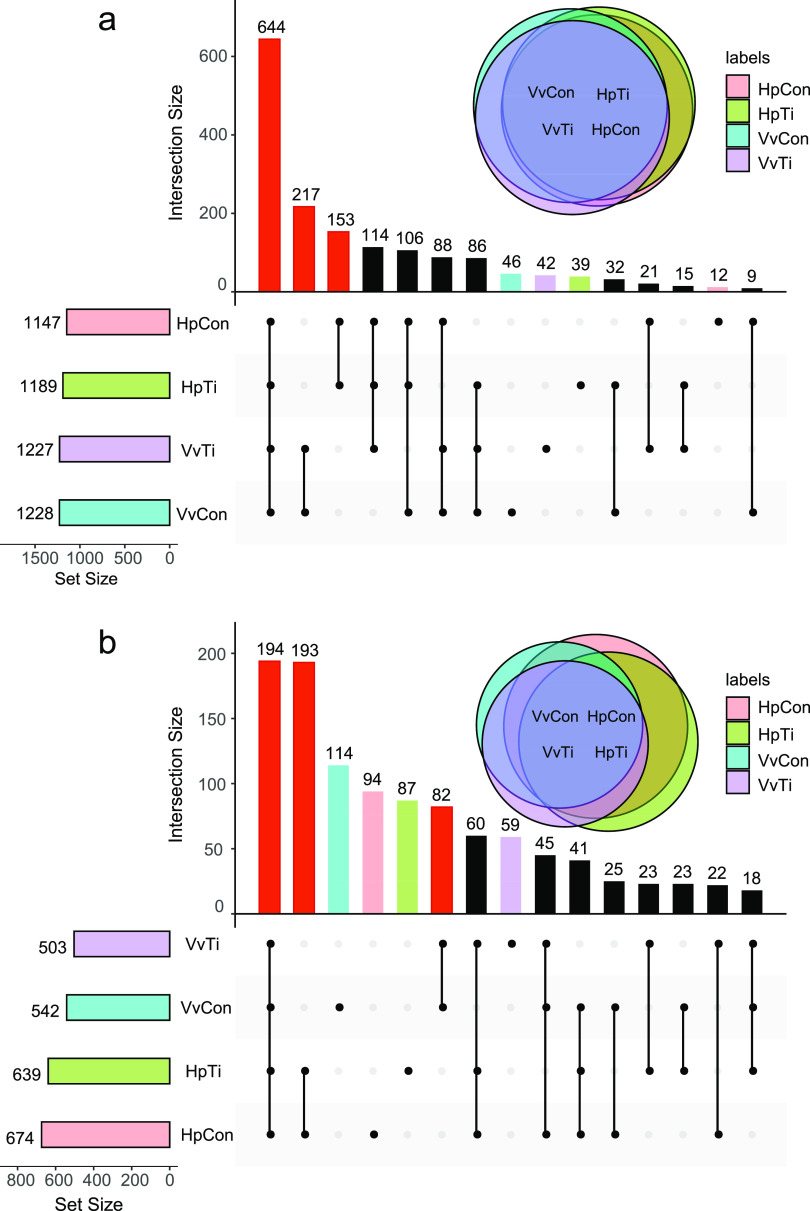
(a, b) Upset and Venn plots of bacterial (a) and fungal (b) communities of the four soil samples. The Upset plot is composed of three parts: the horizontal bars on the left-hand side show the number of OTUs, the exclusive intersections are set (vertically connected filled dark circles) in the center of the plot, and the top vertical bar chart shows the intersection numbers. The different bar colors correspond to the four different groups of soil samples. The specific intersection data are marked in red, while the other intersection data without statistical significance are in black.

The CCS was divided into 1,624 bacterial operational taxonomic units (OTUs) and 1,080 fungal OTUs. Each sample contained 424 to 885 bacterial OTUs and 214 to 450 fungal OTUs. Of the 1,624 bacterial OTUs, 644 were shared among the four samples, 153 were specific to pitaya groups (Hp), and 217 were specific to grape soils (Vv) (Fig. 1a, marked in red). In the bacterial community, the unique OTU numbers for control-treated Hp (HpCon), titanium-treated Hp (HpTi), control-treated Vv (VvCon), and titanium-treated Vv (VvTi) were 12, 39, 42, and 46, respectively. Conversely, of the 1,080 fungal OTUs, 194 were common in all the four soil sample groups, 193 were specific to pitaya, and 82 were specific to grape soil samples (Fig. 1b, marked in red). In the fungal community, HpCon, HpTi, VvCon, and VvTi unique OTU numbers were 94, 87, 114, and 59, respectively. The numbers of common and unique OTUs shown in the Venn diagram are mapped in the histogram in Fig. 1.

### Microbiota diversity.

**FIG 2:**
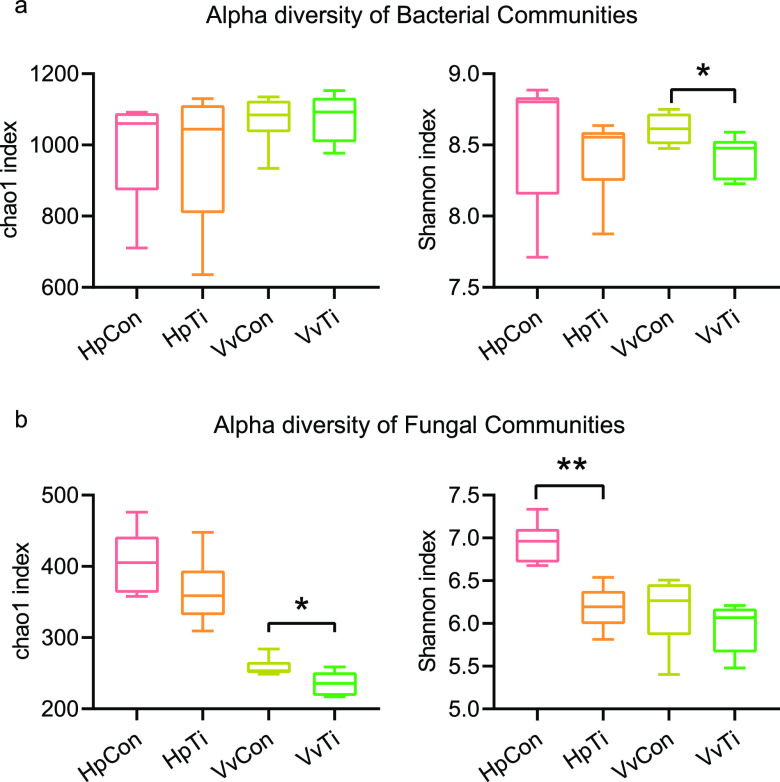
(a, b) Alpha diversity of microbial communities in the soil samples. Chao1 and Shannon indexes of bacteria (a) and fungi (b) are presented. Group differences were determined by Wilcoxon rank sum test (*P* < 0.05); *, 0.01 ≤ *P* ≤ 0.05; **, 0.001 ≤ *P* ≤ 0.01; ***, *P* ≤ 0.001.

The Shannon and Chao1 indexes of bacteria ranged from 7.71 to 8.89 and 635.58 to 1,152.52, while fungi ranged from 5.40 to 7.34 and 217 to 476, respectively (Fig. 2). In the pitaya soil samples, there were no significant differences (*P* > 0.05) in the Shannon and Chao1 indexes of bacterial communities between the HpCon and HpTi groups (Fig. 2a). However, the Shannon indexes of fungal communities were higher in the HpCon group than in the HpTi group (Fig. 2b) (*P* < 0.05). Regarding the grape soil samples, titanium treatment significantly increased the Shannon indexes of bacterial communities and Chao1 indexes of fungal communities (*P* < 0.05).

**FIG 3:**
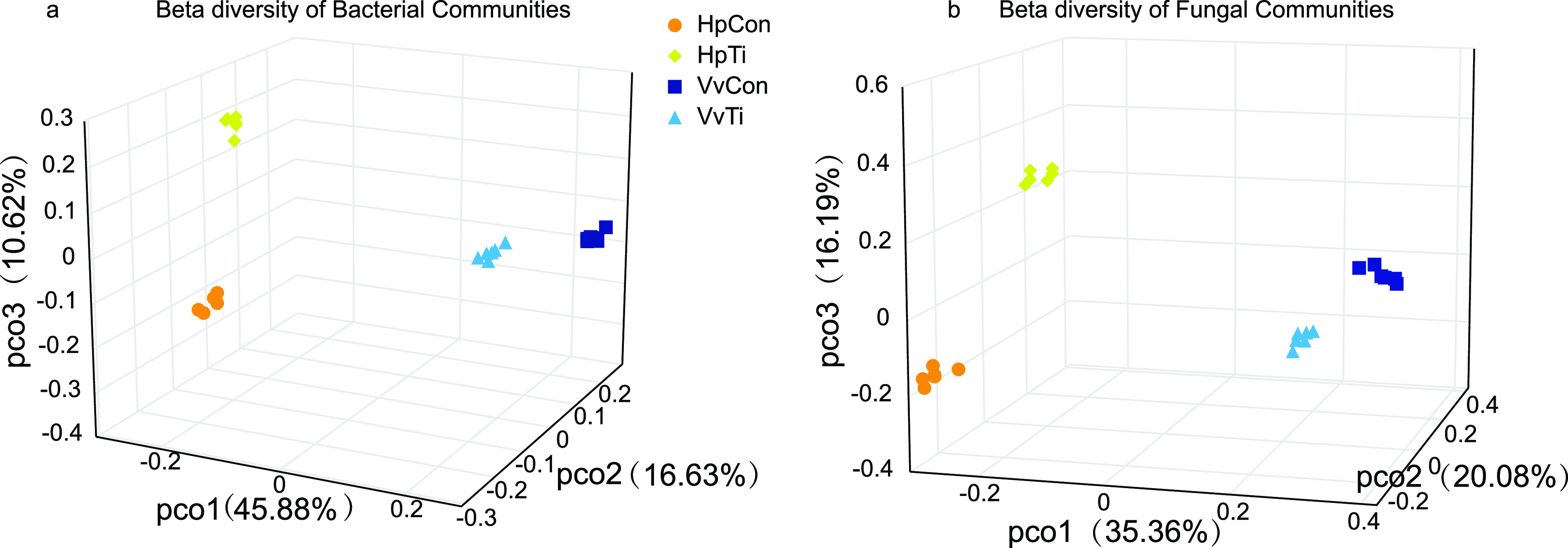
(a, b) Principal coordinate analysis of bacterial (a) and fungal (b) community structures based on Bray-Curtis distance; pco, principal coordinate.

A principal-coordinate analysis (PCoA) showed that the soil microbial community was distinct (Fig. 3). The four groups were separated, and the replicates of each group clustered together. Permutational multivariate analysis of variance (PERMANOVA) showed that the differences between microbial communities were significant (*P* = 0.001) in both bacterial and fungal communities.

### The microbiome structure of bacteria and fungi.

**FIG 4:**
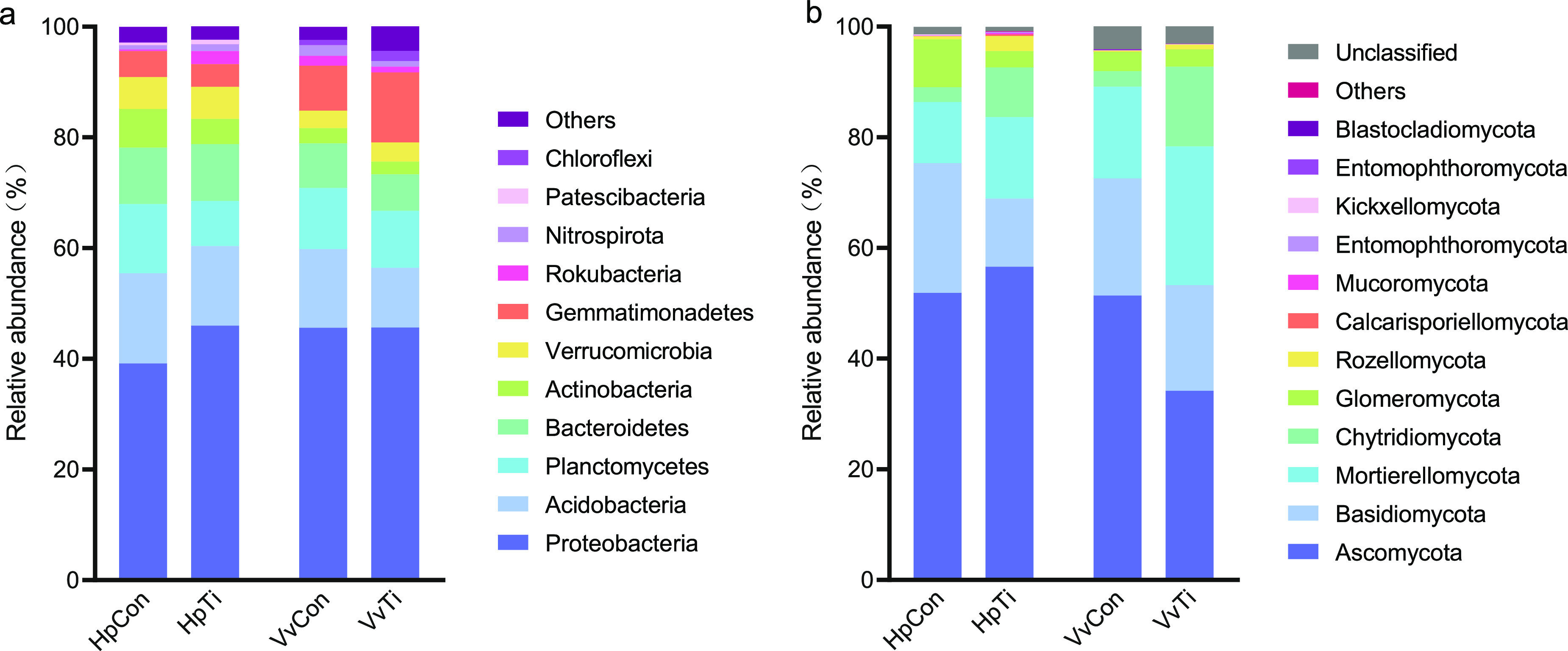
(a, b) Abundance of bacterial (a) and fungal (b) communities in soils planted with pitaya and grape.

**(i) Effect of titanium treatment on the structure of the bacterial community** Titanium treatment significantly altered the structure and richness of bacteria. The bacterial communities in the HpCon and HpTi groups were 25 and 27 phyla, respectively. The seven most abundant bacterial phyla were *Proteobacteria* (mean relative abundance of 39.2% in HpCon and 46.0% in HpTi), *Acidobacteria* (16.3% and 14.4%), *Bacteroidetes* (10.1% and 10.3%), *Planctomycetes* (12.5% and 8.2%), *Actinobacteria* (7.0% and 4.5%), *Verrucomicrobia* (5.7% and 5.8%), and *Gemmatimonadetes* (4.7% and 4.1%) (Fig. 4a). These phyla represented 95.6% and 93.2% of sequences from the HpCon and HpTi, groups, respectively. The relative abundance of *Proteobacteria* and *Rokubacteria* was higher, while *Planctomycetes* was lower in the titanium-treated soils than in the control group (*P* < 0.05).

**FIG 5:**
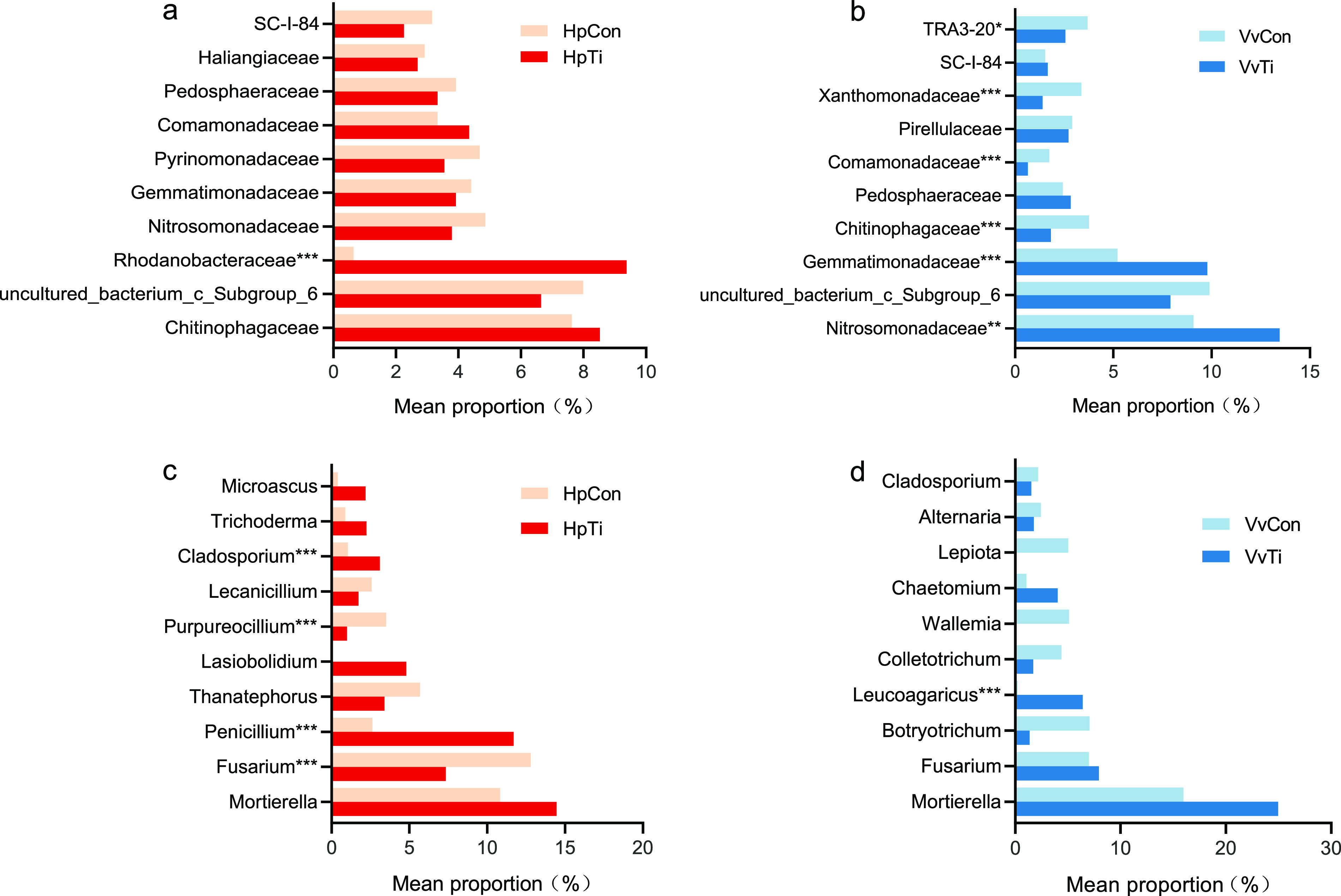
(a to d) Effect of titanium on the relative abundance of bacterial and fungal communities in the soil. The families for Hp (a) and Vv (b) and genera for Hp (c) and Vv (d) are depicted. The number of asterisks indicates significant differences between treatments according to a one-way ANOVA and FDR adjustment (*P* < 0.05); *, 0.01 ≤ *P* ≤ 0.05; **, 0.001 ≤ *P* ≤ 0.01; ***, *P* ≤ 0.001.

A further classification at the family level revealed 24 bacterial families with average relative abundance of >1% in the pitaya soils: 70.8% and 63.6% in the HpCon and HpTi groups, respectively. *Chitinophagaceae* (7.6% in HpCon and 8.5% in HpTi; belong to *Bacteroidetes*), uncultured_bacterium_c_Subgroup_6 (8.0% and 6.6%; *Acidobacteria*), *Rhodanobacteraceae* (0.6% and 9.4%; *Proteobacteria*), *Nitrosomonadaceae* (4.9% and 3.8%; *Proteobacteria*), and *Gemmatimonadaceae* (4.4% and 3.9%; *Gemmatimonadetes*) were the most dominant bacterial families (Fig. 5a). Notably, the relative abundance of *Rhodanobacteraceae* was higher in titanium-treated soils (*P* < 0.001).

Regarding soil samples from the grape field, all the sequences were classified into 27 bacterial phyla. The distribution of the 10 most abundant bacterial phyla is shown in Fig. 4a. *Proteobacteria* (45.6% in VvCon and 45.6% in VvTi) was the most abundant phyla, followed by *Acidobacteria* (14.2% and 10.8%), *Planctomycetes* (11.1% and 10.3%), *Gemmatimonadetes* (8.1% and 12.7%), and *Bacteroidetes* (8.1% and 6.6%). The relative abundance of *Gemmatimonadetes* was higher in the titanium-treated soils, while *Acidobacteria* was significantly lower in titanium-treated soils than in the control group (*P* < 0.05).

A total of 232 bacterial families were identified in soil samples from the grape field. The bacterial communities were dominated by uncultured_bacterium_c_Subgroup_6 (9.9% in VvCon and 7.9% in VvTi), *Nitrosomonadaceae* (9.0% and 13.5%), and *Gemmatimonadaceae* (5.2% and 9.8%) (Fig. 5b). The remaining 20 dominant families with mean relative abundance of >1% accounted for 64.6% and 66.2% of the sequences in the VvCon and VvTi groups, respectively. The relative abundances of *Nitrosomonadaceae* and *Gemmatimonadaceae* were higher in the titanium-treated soils than in the control group (*P* < 0.01). However, the abundance of *Chitinophagaceae* (3.7% and 1.8%; *Bacteroidetes*), *Comamonadaceae* (1.7% and 0.6%; *Proteobacteria*), *Xanthomonadaceae* (3.3% and 1.3%; *Proteobacteria*), and TRA3-20 (3.7% and 2.5%; *Proteobacteria*) were substantially lower in the titanium-treated soil (*P* < 0.05).

**(ii) Effect of titanium treatment on fungal community structure.** A total of 13 fungal phyla were identified in the pitaya-planted soil. The most important phyla accounted for 98.3% of the sequences in each group, including *Ascomycota* (51.9% in HpCon and 56.7% in HpTi), *Basidiomycota* (23.5% and 12.3%), *Mortierellomycota* (11.0% and 14.7%), *Chytridiomycota* (2.7% and 9.0%), *Glomeromycota* (8.6% and 2.9%), and *Rozellomycota* (0.6% and 2.8%) (Fig. 4b). The relative abundance of *Chytridiomycota* was higher in the titanium-treated soils, while those of *Basidiomycota* and *Glomeromycota* were significantly lower in the titanium-treated soils than in the control group (*P* < 0.05).

At the genus level, 237 genera were identified. Of these, 39 genera had a relative abundance greater than 1% in at least one sample, while 22.3% of sequences could not be classified to known genera. The dominant genera of fungal communities were *Mortierella* (10.8% in HpCon and 14.5% in HpTi; belonging to *Mortierellomycota*), Fusarium (12.8% and 7.3%; *Ascomycota*), *Penicillium* (2.6% and 11%; *Ascomycota*), and *Thanatephorus* (5.7% and 3.4%; *Basidiomycota*) (Fig. 5c). The relative abundances of *Penicillium* and *Cladosporium* (1.0% and 3.1%; *Ascomycota*) were higher in the titanium-treated soils than in the control group. In contrast, the relative abundances of Fusarium and *Purpureocillium* (3.5% and 0.99%; *Ascomycota*) were significantly lower (*P* < 0.05) in the titanium-treated soil.

The fungal communities in the grape-planted soil were composed of 11 phyla and other unclassified phyla (3.1% to 4.1%) (Fig. 4b). The five most abundant phyla were *Ascomycota* (51.4% in VvCon and 34.2% in VvTi), *Basidiomycota* (21.2% and 19.1%), *Mortierellomycota* (16.6% and 25.0%), *Chytridiomycota* (2.8% and 14.4%), and *Glomeromycota* (3.5% and 3.1%). The relative abundances of *Chytridiomycota* and *Rozellomycota* were higher while those of *Ascomycota* and *Basidiomycota* were significantly lower in the titanium-treated soils than in the control group (*P* < 0.05).

About 69.8% to 82.0% of sequences were classified into 222 genera at the genus level of fungi. The 10 most abundant genera are shown in Fig. 5d. The communities were dominated by *Mortierella* (16.0% in VvCon and 25.0% in VvTi), Fusarium (7.0% and 7.9%), *Botryotrichum* (7.0% and 1.3%; *Ascomycota*), *Leucoagaricus* (0.22% and 6.4%), *Colletotrichum* (4.4% and 1.7%), *Wallemia* (5.2% and 0), and *Cladosporium* (2.2% and 1.5%). The relative abundances of *Leucoagaricus* and *Penicillium* (0.7% and 1.6%) were higher in the titanium-treated soils, while that of *Dactylonectria* (3.3% and 0.14%) was significantly lower (*P* < 0.001).

### Effect of titanium treatment on the cooccurrence relationships among the bacteria and fungi.

**FIG 6:**
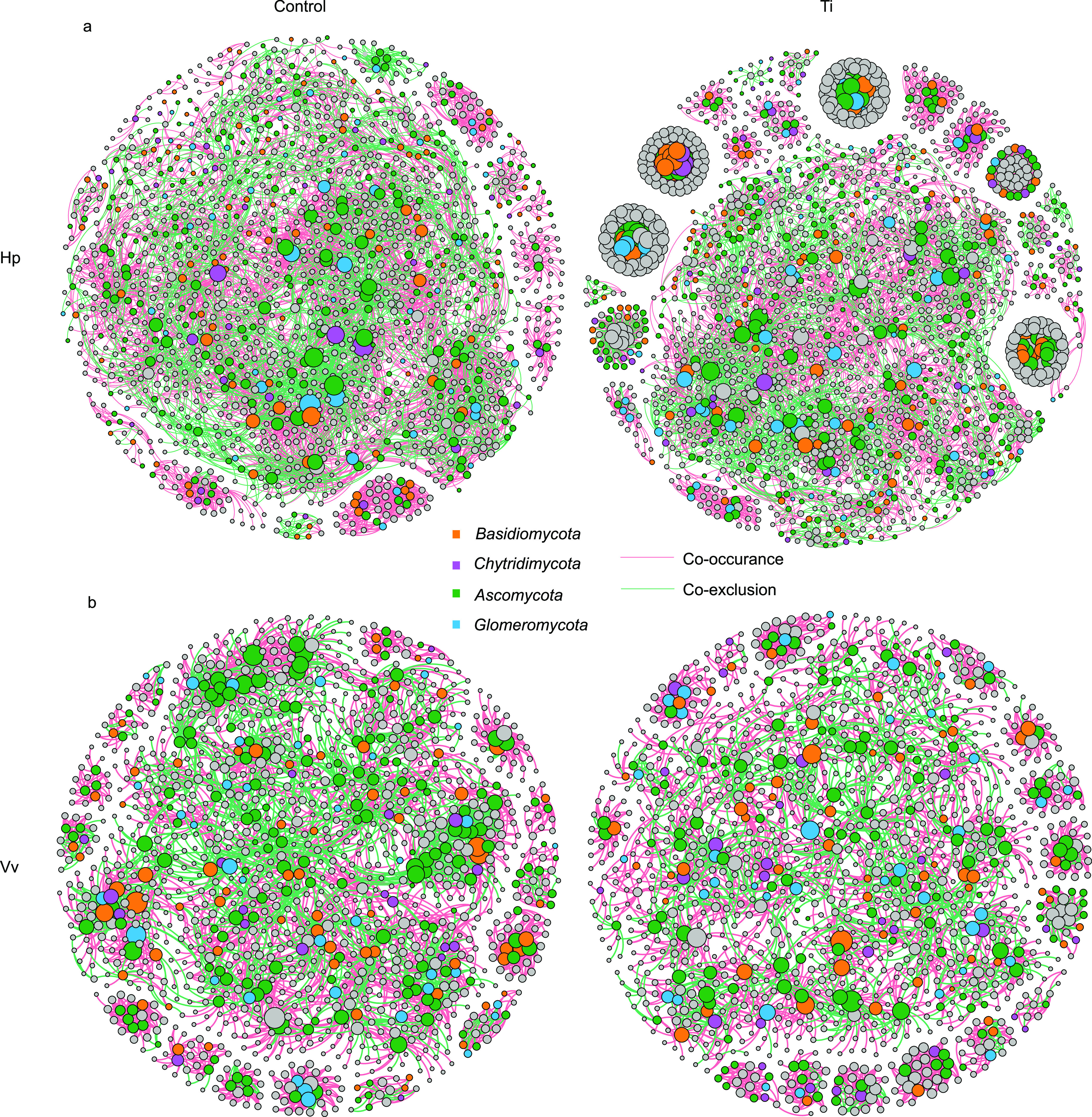
(a, b) Effect of titanium treatment on the bacterial and fungal cooccurrence and coexclusion networks in pitaya and grape agroecosystems. Nodes represent individual OTUs; edges represent significant positive (pink) and negative (green) Spearman correlations (*ρ* > 0.6, *P* < 0.001). Node size is positively correlated with degree (the number of edges attached to a node). Data for HpCon (left), HpTi (right) (a), VvCon (left), and VvTi (right) (b) are shown.

The cooccurrence and coexclusion networks of OTUs in bacterial and fungal soil communities were analyzed at the OTU level (Fig. 6). The network diagram shows 1,804 nodes and 10,961 edges for HpTi, 1,459 nodes and 6,393 edges for HpCon, 1,256 nodes and 3,443 edges for VvTi, and 1,287 nodes and 4,023 edges for VvCon. In the pitaya soil interaction network, the cooccurrence relationship of the HpCon group accounted for 67%, while that of the HpTi group accounted for 81%. Regarding the grape soils, titanium treatment increased the proportion of lines representing cooccurrence from 67% to 70%.

In the pitaya-planted soil, the relative proportion of the cooccurrence relationship was considerably more than the coexclusion relationship, and the cooccurrence was dominant in the interaction network. Fungi with high abundance were important nodes in the interaction network, including *Ascomycota*, *Glomeromycota*, *Chytridiomycota*, and *Basidiomycota*. A cooccurrence relationship exists in the interior of high-abundance phyla and between high-abundance and other low-abundance phyla and between bacteria and fungi.

### Effects of titanium ions on crop susceptibility.

In Panzhihua, pitaya stem rot occurs occasionally, and the infected plants are usually dug out and destroyed to prevent the spread of the disease. The control group had a stem rot susceptibility rate of about 5% in this study, while the treatment group had almost no diseased plants. The disease control measures in grapes mainly involve eliminating powdery mildew, downy mildew, and canker. For grape, fungicide was used three times in the control group and only once in the titanium treatment group. We found that titanium ions could be a safer alternative to farm chemicals.

## DISCUSSION

This study explored the effect of titanium treatment on soil microbial diversity, community composition, and interaction networks. Twenty-four bulk soil samples from a Xichang vineyard and a Panzhihua pitaya orchard were analyzed using PacBio full-length 16S rRNA and *ITS* amplicon sequencing. Notably, this was a field investigation based on the application of titanium ions rather than a designed experiment.

Full-length amplicon sequencing is better at improving the species-level resolution than short-read sequencing (25, 26). However, a long-read sequence may make it difficult to determine the taxa of many uncultured microorganisms in a complex system due to limited existing studies and databases. Herein, the sequences classified as uncultured bacteria were more than half (51.8%) at the genus level and 78.7% at the species level. Regarding fungi, the unclassified sequences were 21.1% at the genus level and 41.4% at the species level. Complex and diverse microorganisms exist in the soil, including many unidentified microbes. Nevertheless, the longer the sequence, the more the information sites and classification accuracy. Thus, full-length 16S rRNA and *ITS* amplicon sequencing exhibit higher taxonomic resolution and have a high potential of discriminating closely related species, relying on more accurate and high-resolution algorithms and continuously updated databases (27).

Evidence indicates that titanium ions have plant growth-regulating activity (15–17, 19). Our previous study showed that titanium ions could improve the expression of genes related to photosynthesis, protein translation, and disease resistance in Medicago sativa (28). Also, it was revealed that titanium ions could prolong the longevity of fresh-cut flowers by inhibiting bacterial growth (19). However, titanium has not been widely applied in agriculture probably because of the lack of in-depth understanding of its impact on soil microbes. This study hypothesized that titanium might influence plant growth by regulating the soil microbial community.

Based on the findings of this study, titanium ions conditioned the soil microbiota diversity. In pitaya and grape fields, the Shannon and Chao1 indexes of bacteria and fungi in the titanium ion treatment groups were lower than those observed in the control groups, indicating that titanium ions reduced the species diversity and uniformity. This is consistent with our previous findings that titanium ions can inhibit bacterial growth and reduce the bacterial diversity in cut flower vase water (19). Although titanium ions inhibited the growth of some microorganisms, the dominant microbes still thrived in abundance. Similarly, it was found that sorghum rhizosphere effects can reduce soil bacterial diversity by recruiting specific bacterial species (29).

The dominant and high abundant microorganisms in the community, also known as the core taxa, or core microbiota, are a group of common microbes in a microbial community that play critical functions in the habitat (30)*. Nitrosomonadaceae* were enriched in the two soil types. Interestingly, *Nitrosomonadaceae* taxa were mainly Ellin6067 and MND1 at the genus level. The two genera were abundant in pitaya soil, but only MND1 was enriched in grape soil. *Nitrosomonadaceae* are nitrifying bacteria that metabolize ammonia (NH_3_) to nitrate (NO_3_^−^), thereby obtaining energy for themselves and providing nitrogen to plants (31, 32). Titanium ion treatment significantly increased the abundance of *Nitrosomonadaceae*, indicating that titanium ions can potentially improve soil nitrogen availability. Furthermore, titanium application significantly increased the relative abundance of *Mortierella*, a dominant fungus. *Mortierella* is effectively used as a biocompound for remediation of heavy metals in soil (33). It is also a potential probiotic involved in regulating the rhizosphere microbial community of plants (34). Thus, the application of titanium ions can potentially increase probiotics beneficial to plants.

In the pitaya-planted soils, the abundance of Fusarium in the titanium ion treatment group (HpTi) was lower than in the control group (HpCon). Fusarium is a potential plant pathogen, causing wilt diseases in many crops, including cotton, watermelon, mango, and banana (35, 36). The genus is associated with significant yield reduction and economic losses worldwide. Inhibition of Fusarium growth by titanium ions probably reduces the susceptibility of crops to related pathogens.

Titanium ion application significantly increased the resistance of the grape and pitaya to various pathogens, thus reducing microbicide application by about half compared with the controls. The drug sensitivity rate of the pitaya control group was about 5%, while there were almost no diseased plants in the treatment group. For grapes, fungicide was used three times in the control group and only once in the titanium treatment group. This indicated that titanium ions could improve plant disease resistance and reduce the use of farm chemicals.

Applying chelated titanium compounds in soil or on plants has increased plant biomass and crop yield (15). Cognizant of this, the application of titanium ions should be considered a safe approach to improving agricultural production. Previous studies on the safety of titanium have also shown that the application of low concentrations of titanium on foliage does not cause the accumulation of titanium in plants (37).

In this study, further analysis of the microbial interaction network indicated that fungi were the critical network nodes in soil microbial associations and may play a vital role in recruitment. It is speculated that titanium ions may affect the whole microbial community by altering the abundance of key microorganisms in the network. Moreover, groups that connect many edges in the network were also present in relatively low abundance, indicating that some microorganisms interact strongly at relatively low abundance. In a complex network microbiome, microorganisms cooperate in providing each other with essential nutrients necessary for growth and survival (38). This cooccurrence is crucial for the stability of the microbial ecosystem (39). Titanium ions promoted the cooccurrence in the network, thus enhancing the stability of the soil microecological structure. It should be emphasized that the interaction networks visualize cooccurrence and coexclusion between taxa but do not necessarily reflect the direct interaction between taxa. Nevertheless, the networks can be used to determine whether the key nodes or abundant species directly affect other microbial members (12). This information can assist in elucidating the specific mechanism of the complex microbial community.

**FIG 7:**
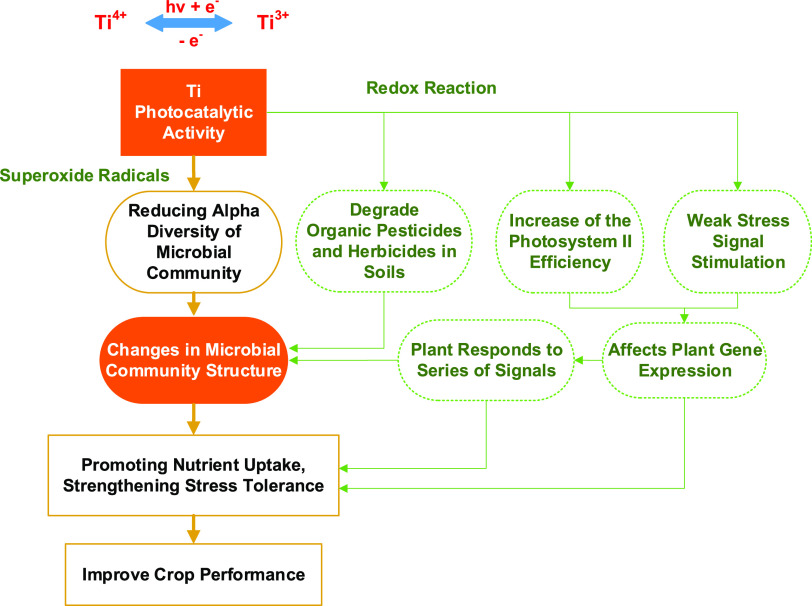
The presumed mechanism of action of titanium ions in improving crop performance. The focus of our research is highlighted in orange, and the arrows indicate the logic of causality.

Based on previous studies and the above results and discussions, we can speculate the mechanism of action of titanium ions (Fig. 7). Titanium has two valence states of Ti^4+^ and Ti^3+^ for electronic energy transition under light. The effect of titanium ions in altering the microbial community is based mainly on its photocatalytic activity, which converts energy from light into chemical energy and triggers redox (40, 41). Redox generates various superoxide radicals, which reduce or oxidize the surrounding medium, potentially degrading soil organic matter, pesticides, and herbicides (42). This changes the soil microecological environment and influences the growth of microorganisms (18). Meanwhile, the photocatalytic reaction directly affects the growth of microorganisms and reduces soil microbial diversity. Moreover, titanium ions directly impact plant growth, which can indirectly affect soil microorganisms. Therefore, future studies should evaluate the interaction mechanism between titanium, microorganisms, and plants.

Beta diversity analysis revealed that the distance between microbial communities of different plants in different areas was larger in the control group than in the titanium ion treatment group. Crop type also has a substantial impact on the structure and function of the soil microbial community (43, 44). Presumably, differences in geographic location and cultivation methods are important drivers of soil microbiome structure (45). The effect of titanium ions on microbial communities varies with different application methods. Therefore, the change in the microbiome did not show convergence. This study used foliar sprays for grapes, while the root irrigation method was selected for pitaya. The method probably significantly impacted the soil microbial community, possibly due to direct contact with titanium ions.

### Conclusions.

The four groups of bulk soil planted with pitaya or grape in Panzhihua and Xichang areas shared a core of bacterial and fungal taxa, including uncultured_bacterium_c_Subgroup_6, *Nitrosomonadaceae*, *Chitinophagaceae*, *Mortierella*, Fusarium, and *Leucoagaricus*. However, the relative abundance of these taxa was different in each soil type. The structural differences between soil and microbial communities of pitaya and grape came from variations in the environment and plant. The application of titanium ions altered the soil microbial communities in different agroecosystems. Titanium ions inhibited or enriched some microorganisms, especially some high-abundant taxa. Although the microbial diversity decreased and the structure of microbial communities was altered, titanium ions enhanced the cooccurrence relationships and improved the stability of the community. The soil microbial interaction network established in this study may provide the basis for further research. However, the specific association mechanism between complex soil microorganisms and agricultural management remains a mystery. This study provides a basis for applying titanium ions in sustainable agriculture.

## MATERIALS AND METHODS

### Site description and experimental design.

This study was conducted in the Panxi region, Panzhihua, and Xichang cities, Sichuan Province, Southwest China. Panzhihua has a humid subtropical monsoon climate, and Xichang has a tropical plateau monsoon climate. The two areas are characterized by abundant sunshine, rain, and concentrated precipitation. The average annual sunshine duration is about 2,500 h, and the average annual precipitation is 1,000 mm.

To prepare titanium ions, antihydrolysis-stabilized ionic titanium (ASIT; patent number PCT/US8308840B2) was provided by Tigrow (Tianjin) Science and Technology Ltd. Titanium ion preparation was performed as described previously (19), with a stock solution concentration of 4,000 mg/L.

Pitaya (*Hylocereus polyrhizus* sp. var. Panxi Dadi-red) crop was used in this study. The crop was planted in 2014 on a farm in rural areas of Panzhihua city (101°72′E, 26°59′N). The field was divided into the control (HpCon) and titanium-treated (HpTi) groups. Root irrigation with diluted titanium ion solution (6 to 8 mg/L) was performed four times on the titanium-treated group, including before the winter of 2018 and at the beginning of spring, flowering stage, and fruit growth stage in 2019. The adjacent control group was irrigated with water. The control and the titanium-treated groups were adjacent to each other in the same field. The soil properties, crop growth environment, humidity, and temperature were similar for both groups. Also, similar agricultural management methods, such as watering and fertilization, were applied in both groups.

The vine (Vitis vinifera L. var. crimson seedless grape) was planted in 2018 on a farm (102°14′E, 27°45′N) in Xichang city. The farm was also divided into treatment (VvTi) and control (VvCon) groups. The vines of the treatment group were foliar sprayed with titanium ions (3 to 4 mg/L) four times, including after winter pruning in 2018 and after germination, flowering, and initial fruiting in 2019. The control group was foliar sprayed with the same amount of water. The soil properties, crop growth environment, humidity, and temperature were the same for both groups. Also, similar agricultural management methods, such as watering and fertilization, were applied in both groups.

### Evaluation of disease control.

In Panzhihua, pitaya has few diseases, but stem rot occasionally occurs. Usually, the infected plants are dug out and destroyed without using microbicides. The incidence rate is calculated by recording the number of destroyed plants. Grape fungal diseases include powdery mildew, downy mildew, and canker. Drugs used for controlling these diseases include Badische Anilin-und-Soda-Fabrik (BASF), Germany kinder 42.4% azole ether fluoramide (2,500 to 4,000 times), Germany Bayer Previcur 722 g/L molamycin hydrochloride (500 to 1,000 times), and DuPont, USA, Kocide 3000 46% copper hydroxide (1,500 to 2,000 times). The health status of the control and the treatment groups was evaluated by the frequency of fungicide use.

### Sample collection.

**FIG 8:**
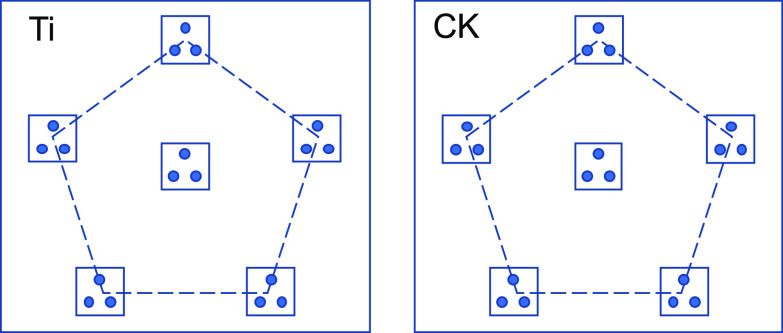
Sampling schematic diagram. CK, control check.

The fields used for the controls and titanium treatments were approximately 40 × 40 m^2^ each. Titanium was applied to all plants in the Ti-treated field, while all the control plants were not treated. Briefly, six sampling points (5 × 5 m) were randomly selected in each group. Triplicate soil samples from each point were taken (10- to 20-cm bulk soil layers) and mixed. Six sampling plots (5 × 5 m) were established for each treatment before sample collection. Each sampling point was marked in a triangular manner denoted by three dots (“•”), as shown in Fig. 8. From each point, six equally spaced repetitions were performed for a total of 24 samples. All the bulk soil samples were collected in November 2019. Fresh soil samples were sieved with 2-mm mesh to remove rocks, plant residues, and impurities and then divided into two parts. Thereafter, one part was used for DNA extraction, while the other was stored at −80°C.

### DNA extraction and amplicon sequencing.

Total DNA was extracted from each soil sample using the PowerSoil DNA isolation kit according to the manufacturers’ instructions (MO BIO Laboratories Inc., CA, USA). The isolated DNA samples were diluted 10 times, and a SynergyHTX enzyme labeled instrument (Gene Company Limited) was used to check the DNA quality and quantity, according to the manufacturer’s instructions.

Microbial diversity analysis was performed based on the 16S rRNA gene for bacteria and the internal transcribed spacer (*ITS*) region for fungi. Bacterial and fungal total genomic DNA were amplified using primers specific to the full-length bacterial 16S rRNA region (46) (27F: AGRGTTTGATYNTGGCTCAG; 1492R: TASGGHTACCTTGTTASGACTT) and the full-length fungal *ITS* region (47) (ITS1: F-CTTGGTCATTTAGAGGAAGTAA; ITS4: R-TCCTCCGCTTATTATTATGC). The forward and reverse primers were tailed with the sample-specific PacBio barcode sequence for multichannel sequencing of each sample.

PCR products were quantified from the gel images using ImageJ. The amplicons were recovered from the gel and purified using 0.8× magnetic beads (MagicPure size selection DNA beads, TransGen Biotech). High-throughput sequencing was performed using the PacBio Sequel platform at Biomarker Technologies Company (Beijing, China).

### Data processing.

Circular consensus sequences (CCSs) were obtained by correcting the original subreads (SMRT link, version 8.0) according to minPasses of ≥5 and minPredictedAccuracy of ≥0.9. Lima v1.7.0 software was used to identify different samples according to the barcode. Cutadapt V2.7 (error rate 0.2) was used to identify the forward and reverse primers, and CCSs without a primer were discarded. Finally, CCS length was filtered, and sequences that did not meet the length threshold (16S: 1,200 bp to 1,650 bp; ITS: 300 bp to 1,000 bp) were discarded.

Usearch (48) software was used to cluster CCSs at 97% similarity level, obtain operational taxonomic units (OTUs), and conduct taxonomic annotation of the OTUs based on Silva 132 (bacteria) (49) and UNITE 8.1 (fungi) (50) taxonomy databases. Query sequences were blasted against the reference database using the classify-consensus-blast methods, and the nonmatched sequences were further classified by classify-sklearn. Data on the community composition of each sample was obtained at various classification levels (phylum, class, order, family, genus, and species) and analyzed to generate species abundance tables using QIIME software (51).

The alpha diversity index of each sample was evaluated using Mothur (version v. 1.30) software (52). Chao1 indexes and Shannon indexes were used to determine the microbial diversity of each sample.

Principal coordinate analysis (PCoA) was performed using the OmicShare tools (http://www.omicshare.com/tools). Upset plot analysis of OTUs was performed using the OmicStudio tools (https://www.omicstudio.cn/tool).

### Network analysis.

To further explore the effect of titanium application on the occurrence and coexclusion patterns of soil bacterial and fungal communities, we performed Spearman rank correlations (R version 3.6.1) on the relative abundance of OTUs at all groups (*ρ* > 0.8, *P* < 0.001). All networks were visualized with the Fruchterman-Reingold layout permutations in igraph. Correlation analysis was performed using the OmicStudio tools at https://www.omicstudio.cn/tool/62.

### Statistical analyses.

A Wilcoxon test was used to determine the differences in alpha diversity among different groups. A permutation multivariate analysis of variance (PERMANOVA) was performed based on different matrices to determine whether beta diversity differed significantly between the groups. Differences in the relative abundance of phyla and family/genera were determined using a one-way analysis of variance (ANOVA). Storey false-discovery rate (FDR) was used to determine the *P* value threshold in multiple tests and analyses. A *P* value of ≤0.05 indicated a significant difference.

### Data availability.

PacBio sequence data for 16S rRNA and *ITS* genes are available in the NCBI Sequence Read Archive (SRA) under the BioProject ID PRJNA699417.
